# Enhancing the resilience of remote patient monitoring and hospital-at-home systems: a digital-twin-based framework

**DOI:** 10.1038/s44401-026-00109-9

**Published:** 2026-07-01

**Authors:** Max Ostermann, Anne Sophie Platzbecker, Cindy Welzel, Stephen Gilbert, Oscar Freyer

**Affiliations:** 1 Else Kröner Fresenius Center for Digital Health, TUD Dresden University of Technology, Dresden, Germany; 2 Hasso Plattner Institute, Potsdam, Germany; 3 Medizinische Universität Lausitz—Carl Thiem, Cottbus, Germany; 4 Faculty of Business and Economics, TUD Dresden University of Technology, Dresden, Germany

**Keywords:** Engineering, Health care

## Abstract

Remote patient monitoring (RPM) enables clinical oversight of patients outside the hospital via connected devices and supporting digital infrastructure. Hospital-at-home (HaH) is a high-acuity specialization within RPM. As RPM programs expand in scale and acuity, they face significant challenges to resilience that can compromise patient safety. We propose integrating simple patient Digital Twins (DTs), which are personalized models continuously updated with real-time data, to enhance the resilience of both HaH and RPM systems by supporting informed clinical management even when primary systems are unavailable, thereby improving patient safety during periods of network or other system unavailability.

## Introduction

Advances in sensor and information technology have given new momentum to a care concept that was first mentioned and tested in the 1970s: the hospital at home (HaH)^1^. This concept, also referred to as ‘virtual ward’, is defined as delivering care in a patient’s home environment with an intensity similar to that provided in a hospital^2^, through means such as remote patient monitoring (RPM). While the HaH is a high-acuity specialization within the broader RPM concept, the failure modes and resilience requirements discussed here apply broadly to RPM services. Traditionally, healthcare delivery in the patient’s home relied on the physical presence of healthcare professionals (HCPs) providing in-person care^1^. However, advancements in technology have enabled an asynchronous system where direct patient-HCP interaction is infrequent, and digital technologies such as RPM systems and network-connected sensor technology in and around the patient, referred to as the Internet of Medical Things (IoMT), are utilized. The HaH model has proven a satisfactorily safe concept^3–7^, providing high-quality care to patients with acute and chronic diseases^8^ as well as to high-acuity patients^9^ at a lower cost but with potentially better outcomes, e.g., due to faster recovery and reduced rates of complications like infections^1,5^.

At the same time, the dependency of those services on digital systems and network communications raises concerns about the security and reliability of such systems in a world with an increasing number of cyber threats against healthcare systems (e.g., cyberattacks and network outages)^10,11^. Attacks could be employed via multiple vectors and can affect a HaH service in three different ways: a loss of privacy with disclosure of patient data, the modification of patient data, and the disruption of the service^12^. Healthcare data is highly sensitive to a patient’s privacy, and in real-time monitoring, the integrity and availability of data can be critical to a patient’s health. However, it is becoming increasingly difficult to guarantee all three aspects, as shown by an increase in cyberattacks on hospitals over the last years^13^. While the privacy problem is already discussed in the literature^1^, there is currently a gap in how the other two risks should be addressed, although the consequences of such an attack could pose major risks to the health of affected patients, e.g., delayed detection of clinical deterioration or impaired triage and prioritization of high-acuity patients. Existing research on patient DTs in healthcare has focused largely on precision medicine, organ- or disease-specific simulation, and predictive applications in oncology and clinical trial design^14–17^. Recent work has begun to look at DTs in HaH care, mostly at the level of the individual patient^18^, and at DTs as a threat detection tool inside hospital Internet of Things (IoT) environments^19^. The idea of using DTs as a mitigation for service disruption in distributed RPM and HaH care has not been explored in either strand. Our framework draws on the same underlying DT concept but applies it to a different problem: maintaining clinical oversight when the supporting infrastructure is partially or fully unavailable.

On the one hand, it can be assumed that attacks and non-malicious incidents will occur in HaH systems as soon as these systems are widely used. On the other hand, acceptance also depends on trust in and resilience of such systems. In the cybersecurity context, per the definition of the National Institute of Standards and Technology (NIST), resilience refers to the ability of a system to anticipate, withstand, recover from, and adapt to adverse conditions, attacks, or compromises on its resources^20^. This has already been recognized by legislators, who have issued rules to make healthcare infrastructure more secure and resilient^21–23^. However, existing resilience and cybersecurity strategies for RPM and HaH systems primarily aim to prevent or mitigate system failures, but do not address how clinical oversight and patient prioritization can be maintained once system availability is partially or fully lost. In this paper, we describe how RPM and HaH systems are vulnerable to cyber threats and propose a framework to enhance their resilience by utilizing ward-level digital twins (DTs) for risk-scoring purposes, as well as present an outlook on how patient-specific DTs and artificial intelligence (AI)-based predictive health approaches might increase the resilience even further.

### Systemic vulnerabilities in RPM and HaH infrastructures

HaH describes a concept where patients who require inpatient admission and therapy receive all necessary treatments at home, in some cases, even fully preventing the need for hospitalization. Thus, even for patients with critical diseases, medical care is delivered in a patient’s home environment with an intensity similar to that provided in a hospital^24^. The concept received a lot of attention during the COVID-19 pandemic^25,26^, and is now seeing continued research and adoption^1,6,27,28^, as well as being described as part of the “future of healthcare”^29^.

**Fig. 1:**
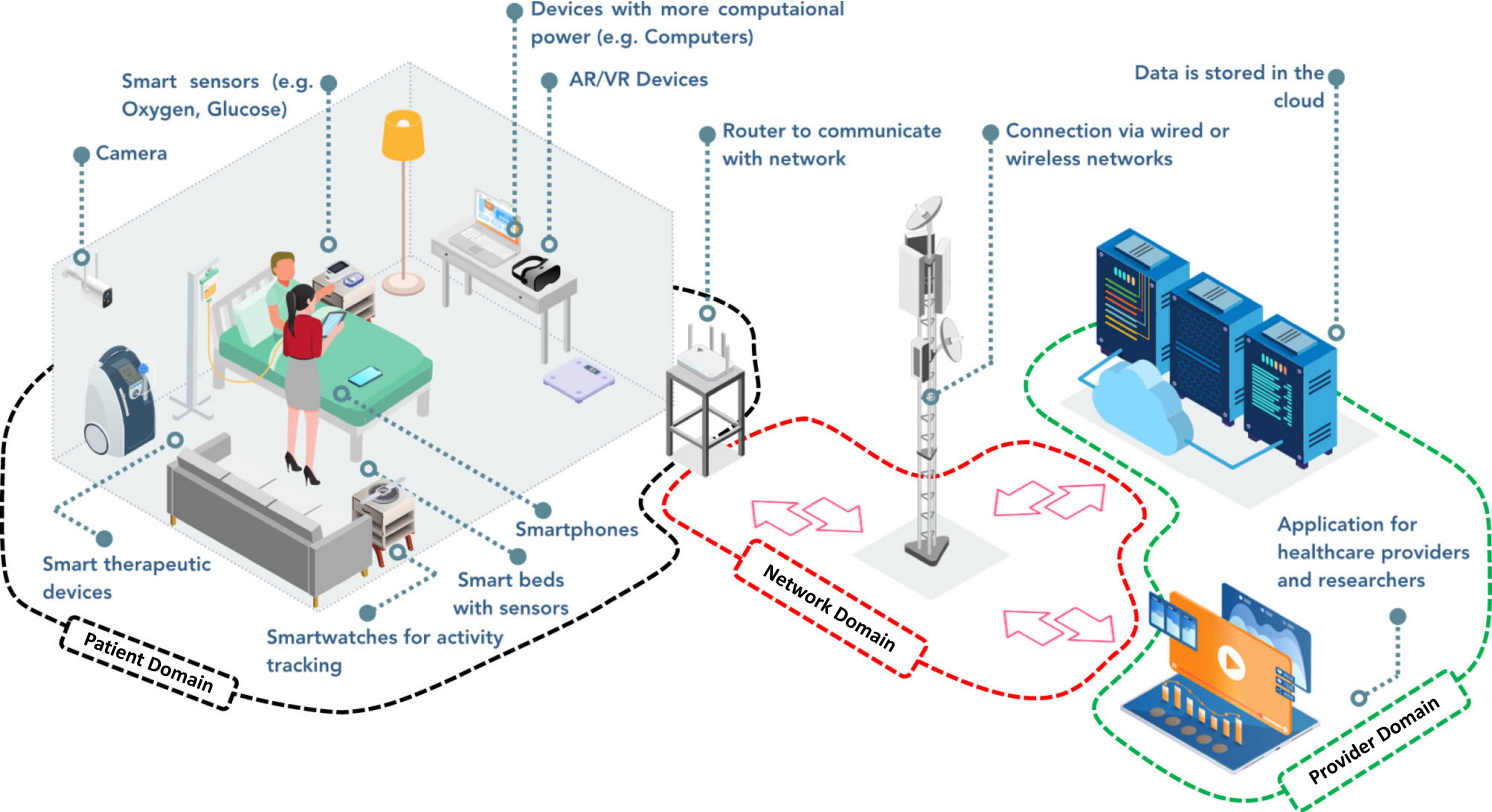
Depiction of a HaH system*.* An example of what a HaH setup could look like, showcasing a number of different components.

Modern RPM and HaH infrastructures usually consist of multiple hardware and software devices distributed over three domains. In the patient’s home domain, the clinical state and health-related parameters such as pulse, oxygen saturation, or blood pressure are constantly monitored with IoMT devices^1,27,30^. In the network domain, the data gathered in the patient’s home is transferred to the provider using cable or cellular network connections^30^. In the HaH provider domain, the data is reviewed, analyzed, and stored to provide real-time monitoring of the patient. Here, modern concepts facilitate cloud computing for the handling of large amounts of data and AI-based analysis and prediction^30^. Regular in-person visits ensure the safety of patients in HaH systems as they allow the detection of subtle clinical changes that might not be captured digitally and to intervene promptly when necessary. If the system registers a deterioration of a patient (e.g., an anomaly in an electrocardiogram), countermeasures are initiated, such as advising the patient on self-treatment, examination of the patient through a telemedicine service, dispatching of mobile medical teams or specialized emergency physicians to the patient’s home, or informing the emergency services to provide treatment in a healthcare facility. Figure 1 provides an overview of the components of such a system.

The fundamental dependency on digital connectivity in HaH systems creates a critical vulnerability: the risk of service interruption. Consequently, the impact of the primary scenario this research aims to reduce is the loss of system availability, effectively a Denial of Service (DoS) state. This state can occur across all three domains (patient, network, and provider) and results in the inability to transmit or process vital patient data^31^. While literature specifically regarding HaH security is emerging, findings from RPM and EHR systems confirm that maintaining availability is the central challenge in ensuring patient safety^12,32^.

This state of service denial can be triggered by two distinct categories of threats. The first is malicious cyber activity, specifically DoS attacks, such as flooding or spamming attacks, which aim to overwhelm the network bandwidth or storage capabilities of the service^12,32^. The second category encompasses non-malicious incidents, such as network infrastructure failures, natural disasters, or human error. Although often categorized separately, both attack vectors result in the same “exact scenario”: a critical failure of the HaH system’s availability. Table 1 provides an overview of these targets and the specific vectors that lead to these disruptions. While cybersecurity mechanisms can reduce the surface area for and probability of malicious attacks^12^, they cannot eliminate the risk of outages caused by physical or operational failures^33^.

**Table 1:** Exemplarized cyberattack vectors in the three domains of HaH systems

Domain	Patient	Network	Provider
Infrastructure/devices of interest	Networked actuators or sensors (e.g., blood pressure monitor or insulin pump)	Patient’s network or gateway	Provider’s network/databases
Attack target	Data collection	Data transmission	Data storage
Confidentiality	Unauthorized access to the device	Man-in-the-middle	Unauthorized access/data breaches
Integrity	Selective forwarding, desynchronization, malware	Modification	Malware
Availability	Scrambling, flooding, jamming	Scrambling, allocation, wormhole (DoS)	Malware, DoS

The attack vectors are categorized by attack domain and impacted CIA property.

The impact of these disruptions ranges from temporary data gaps to a complete cessation of care services, with a set of possible consequences provided in Table 2. A crucial distinction must be made regarding the nature of the failure: while a loss of availability (system outage) is typically immediately detectable, attacks targeting data integrity pose a mo 2 re insidious threat. If a threat actor bypasses availability disruptions to instead manipulate patient data undetected, it could lead to incorrect clinical decisions and long-term patient harm.

**Table 2:** Consequences of attacks

Domain	Patient	Network	Provider
**Confidentiality**	Exposure of sensitive health data from compromised IoMT devices (e.g., blood pressure monitors, insulin pumps).	Data interception during transmission, e.g., through Man-in-the-Middle attacks, potentially leaks personal and clinical data.	Breaches of backend databases leading to massive data leaks and violations of privacy regulations (e.g., GDPR, HIPAA).
**Integrity**	Alteration of health readings (e.g., forged oxygen levels), leading to misinformed clinical decisions and possible patient harm.	Injection or modification of data in transit (e.g., false telemetry), compromising treatment decisions due to erroneous health trends.	Tampered records in clinical databases (e.g., medication records, alerts), causing long-term clinical and legal complications.
**Availability**	Device jamming causes devices to become unavailable, preventing vital data from being transmitted.	Disruption of the communication infrastructure leading to system-wide data unavailability.	Downtime in central systems due to ransomware or DoS attacks affecting scheduling, alerting, decision-support systems, and storage/retrieval of EHRs^36^.

The attacks’ consequences are categorized by attack domain and impacted CIA property.

Current HaH systems are beginning to be rolled out. While up until recently HaH systems were only used by a small number of patients in research or testing scenarios^3,5,8^, wider rollouts have begun in countries like the US, Switzerland, Israel, and the UK^34^. This suggests that HaH services will soon become broadly available, including for patients with severe conditions^35,36^. In a scenario where a high number of patients are dependent on the HaH system, the consequences of a loss of functionality could be dire^36^. Patients with critical conditions rely on continuous monitoring and timely interventions, and a system outage would leave them vulnerable.

A loss of HaH service availability—whether due to adversarial attacks (e.g., on the network connection) or technical errors (e.g., network outages)—puts every remotely monitored patient at risk, as health deterioration cannot be recorded and timely interventions cannot be initiated. In such a systemic failure, the only failsafe is to physically check on all monitored patients through in-patient visits or checks via telephone. Given the theoretically possible number of monitored patients, this uniform reliance on in-person checks is infeasible within a reasonable timeframe due to limited resources, which could lead to serious harm or death of patients. Consequently, without access to real-time data or an alternative prioritization method, doctors would face significant challenges in triaging care. This high level of personnel required to mitigate the associated risks with the available resources could inhibit the widespread adoption of HaH models, as the expected cost reductions cannot be realized here, and the existing models cannot be scaled up.

The implementation also differs between approaches to the device suites in a HaH. While some systems have opted for a model that ships a pre-configured suite of devices to the patient, the FDA has proposed a more flexible approach^37^. They have launched a framework that explores the inclusion of consumer devices in RPM/the HaH, which would allow a “bring-your-own-device” (BYOD) approach. While BYOD allows for RPM and HaH systems to be more flexible and convenient for patients already in possession of some suitable devices, it also introduces new threats to the network^38^. This is especially relevant, as, unlike strictly controlled IT perimeters in hospitals, home networks also host other consumer devices that could enable lateral movement, exploring the network for further exploits after an initial breach.

At the same time, the threat landscape for healthcare infrastructures such as HaH systems is rapidly evolving. As highlighted by the European Commission’s 2025 “Action Plan on Cybersecurity in Hospitals and Healthcare Providers”^39^, healthcare systems are increasingly targeted by financially motivated criminals, ideologically driven actors, and state-sponsored campaigns^23^.

This growing risk environment not only challenges the secure operation of RPM/HaH systems but could also undermine public trust and jeopardize their broader implementation, as risk minimization is also a regulatory requirement^33,40–44^. To address these trends and build resilient infrastructures, traditional best practices^45,46^ like network segmentation^47^ are enhanced with approaches such as automated vulnerability scanning, IoT patching, or tunneling for unpatchable legacy devices^48^, and zero trust architectures. These become especially important if the suites of devices are not managed by a central party, but by the patients themselves or by individual third parties in BYOD settings. Loss of availability is a predictable failure mode and needs to be addressed.

### Digital twins as a framework for risk mitigation and resilience

DTs are defined as sophisticated simulations of existing real-world entities that are constantly updated with real-time data^49^, already used in multiple sectors^50–52^. In the healthcare sector, this concept currently means patient-level virtual and interactive representations of organs or patients, which are constantly updated with data from various sources like IoMT sensors, wearables, and environmental sensors^14,53^ and are already part of the HaH system architecture. This representation, the DT, can then be used for multiple applications, including in precision, personalized, and predictive medicine, clinical trials^14–17^, medical device and drug design^14^, and healthcare management^14^. The granularity proposed in this framework is a domain-specific ward-level DT, where the domain is each patient’s unique virtual ward itself. It operates on the aggregate of all patients enrolled in the ward and links their historical records and real-time data streams to derive continuously updated risk scores and prioritization information^14^.

We argue that the real-time updating and prediction functionalities of simple domain-specific ward-level DTs could serve as a basis for improving the reliability of patient safety oversight in HaH systems by providing a means of appropriately targeting coordination and management of action and response activities needed in the case of a major service disruption. The NIST framework for cyber-resilient systems identifies key capabilities that resilient architectures should support - the ability to anticipate adverse conditions, withstand them while maintaining essential functions, recover from disruption, and adapt to evolving threats^20^. The ward-level DT contributes to three of them. Continuous risk scoring during normal operation supports anticipation by monitoring the overall system state and surfacing patients whose trajectories are trending toward deterioration before an incident occurs. During a loss of availability, the DT’s last-known state and associated risk scores support withstanding the disruption by enabling targeted triage rather than uniform in-person checks. Over time, outcome feedback supports adaptation by allowing the underlying risk model to be recalibrated as threats and patient populations evolve.

Although sophisticated patient-specific DTs for diagnostic and treatment planning purposes remain far from readiness for use in everyday care^54^, we point out how DTs at the level of the virtual ward could be developed to better define the allocation of assistance in the event of HaH disruption—it is critical that in such a scenario the limited available medical personnel are effectively targeted to patients most in need of emergency attention.

Domain-specific ward-level DTs could address this need through virtual ward-specific modeling, linking historical status information of all patients in a ward to sensor outputs as they correlate with overall patient health status and health deterioration, and interpreting these to provide prioritization information when live data becomes partial, interrupted, incomplete, or unavailable. Right now, this information could be used to create a live risk-score with near-future prediction capabilities for every patient in the virtual ward of a HaH, informing the triage for in-person visits^55–57^. Future implementations are likely to be able to predict patients’ health trajectories over a longer time through patient-specific DTs^58^. At present, such patient-specific DTs should be understood as a longer-term research vision, whereas ward-level DTs focused on risk scoring and prioritization represent a more immediately feasible and regulatory-compatible approach.

### Risk scoring using digital twin-enhanced HaH systems

Conceptually, the risk score draws on familiar inpatient scoring approaches such as the Rothman Index, which continuously aggregates routinely collected clinical data such as vital signs into a single measure of patient condition^59^. A ward-level DT extends this principle to the home environment, deriving a comparable score from IoMT, wearable, and patient-reported data streams. The specific input set and aggregation function will need to be determined empirically.

**Fig. 2:**
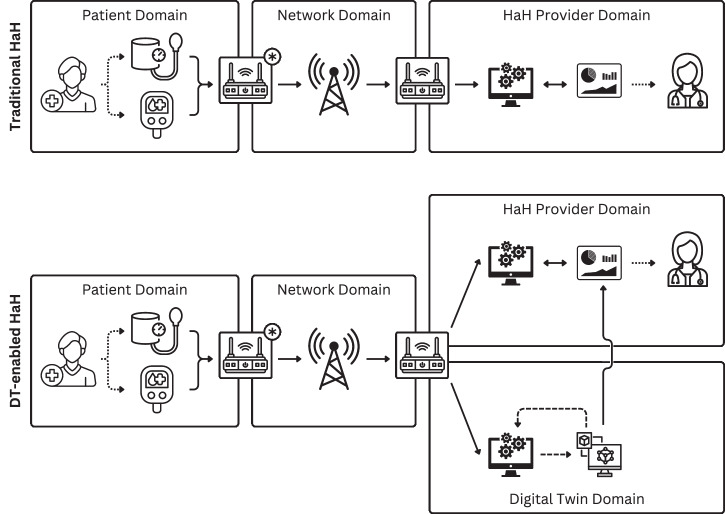
Infrastructure of a DT-enhanced HaH System*.* An overview of the different domains a DT-enhanced HaH system covers. *Optional, as devices could also transfer their data directly.

Building on this principle, implementation of a DT-enhanced HaH system could be realized following a multistep approach. Firstly, when a patient is admitted to a virtual ward, an initial risk score is created using existing health data records, as well as static acuity levels and fed into the ward-level DT. Secondly, the DT monitors incoming real-time data and recalculates the risk score for every patient enrolled in the virtual ward. The resulting risk score is intended to support clinical decision-making and prioritization by HCPs, rather than to trigger automated clinical decisions or interventions. Current concepts of HaH systems already fulfill all requirements of ward-level DTs, since real-time monitoring, real-time data streams, as well as different data sources, are already provided, and no additional data is needed for the creation of the risk score and the DT. Figure 2 shows how the ward-level DT is integrated into the HaH service. Table 3 shows possible input parameters and their roles in the DT.

**Table 3:** Components and data flows of the ward-level Digital Twin

Component	Source	Role in the DT	Contribution to output
Admission baseline	Electronic health record, intake assessment	Static reference capturing diagnoses, comorbidities, acuity level, and medication regimen at the time of enrollment	Establishes the initial risk score and the reference envelope against which live data is compared
Historical patient data	Prior HaH episodes; aggregated ward-level outcomes	Provides population-level patterns linking sensor trajectories to deterioration events	Calibrates the scoring function; informs the expected-trajectory model used for anomaly detection
Real-time physiological data	IoMT devices, wearables (e.g., pulse, SpO₂, blood pressure, ECG, and activity)	Continuously updates the patient's current state representation	Drives recalculation of the risk score and near-future trajectory prediction
Patient-reported inputs	Symptom check-ins, app-based questionnaires	Captures clinically relevant signals not measurable by sensors (e.g., pain, dyspnea, adherence)	Supplements the physiological data in the score calculation
Contextual factors	Admission record, care-team logistics (living situation, caregiver presence, home accessibility, distance to response team)	Characterizes the feasibility and timeliness of in-person response	Refines prioritization output to reflect both clinical urgency and reachability
Home factors	Individual configuration of each home, device set-ups, and network configurations	Models each patient's home as a single-patient room within the virtual ward, capturing the home’s state alongside the patient’s state	Informs anomaly flags by setting per-home baselines for expected device and connectivity behavior
Outcome feedback	Clinical notes, intervention records, and disposition outcomes	Retrospective signal on whether prior scores correctly predicted the need for intervention	Supports calibration and adaptation of the scoring model over time
→ Riisk score	Output	Continuously updated per-patient score analogous in concept to the Rothman Index	Primary input to HCP decision-making on level of care
→ Prioritization ranking	Output	An ordered list of patients reflecting combined clinical urgency and response feasibility	Guides the allocation of in-person visits, particularly during service disruption
→ Anomaly flags	Output	Alerts where incoming data deviates implausibly from the patient's own historical envelope or from multi-sensor consistency	Supports integrity cross-checking and surfaces candidate data-quality issues

Overview of the input sources, processing functions, and outputs of the ward-level DT, illustrating how heterogeneous data is integrated into continuously updated risk scores and prioritization information. Specific input parameters and the form of the aggregation function are use-case dependent and would need to be determined empirically.

The real-time risk scores with near-future prediction enable HCPs to make decisions about the required level of care for each patient, which patients need to be treated in person or need to be taken back into the hospital, and estimate the need for emergency services. Personnel could be coordinated and distributed more effectively, reaching patients who actually require in-person care more effectively, while not wasting resources on patients who do not need this level of care. During a service disruption, prioritization would not be determined by clinical risk alone but also by the timeliness of in-person response and other factors. The ward-level DT could incorporate non-clinical factors routinely captured at HaH admission, including whether the patient lives alone, the presence of informal caregivers, the individual ward set-up, the accessibility of the home, and the travel distance from available response teams. Combined with clinical risk, these contextual inputs would enable more effective allocation of limited medical personnel than a pure physiological score would allow.

Should further advances in the development of DTs and in predictive health be successful, the described and currently feasible ward-level DT system for enhanced resilience could hypothetically be improved by implementing patient-specific digital twins with the capability of advanced predictions of patient deterioration. It would be able to provide assistance in identifying which patients can safely remain under simulated observation and which require immediate in-person attention to enable a risk-aware distribution of limited clinical resources. Or, in even further improvements, treat acute-on-chronic disease events autonomously, when a suite of devices in a HaH can both diagnose patients and administer a number of predetermined, likely to be necessary medications, via an autonomous smart pump, as well as flagging anomalous data that probably stems from issues of data integrity rather than actual measurements of the patient.

### Implementation challenges: technical, ethical, and regulatory considerations

However, before the implementation of DTs into HaH systems is possible, several challenges need to be addressed. From a technical perspective, DT integration requires interoperability across diverse data sources and device types, which is particularly demanding in BYOD settings where device suites are heterogeneous and not centrally managed^38^. Validation frameworks for DTs are still emerging, and standardized methods for verifying that a DT faithfully represents its physical counterpart remain an open research area^60,61^. Another challenge is the reliance on the integrity of the data that the DT receives. Tampering with the data during transmission is less likely to be an issue, as this can be addressed through a number of cryptographic protocols, such as signatures. However, deteriorating sensors that measure data incorrectly would be an issue for the model, especially if the deterioration is probable and unlikely to be detected as an anomaly. Ethically, patients must provide informed consent and be aware of how their data will be used^62–64^, including that the DT may inform prioritization during periods of degraded system availability. Algorithmic bias is a further concern: systematically lower scores for certain subgroups could translate into delayed care during outages, making bias a patient-safety issue in this context rather than only a fairness concern^65^. Accountability when a DT-informed triage decision contributes to harm needs to be articulated before clinical deployment.

From a regulatory perspective, DT-based risk scoring is best understood as a clinical decision support function on a patient level and an AI-related product safety feature for the HaH, with final responsibility remaining with HCPs. Developers are required to implement risk-management strategies that reduce patient risk to an acceptable level^23,33,41–44,66,67^. Additional requirements for cybersecurity and resilience exist for healthcare infrastructure^40,68^, AI applications^67^, and data spaces such as the European Health Data Space^69^. Approval as medical devices will require DT-specific validation frameworks, building on approaches proposed for other AI-based systems^60,61^. Beyond these, the resilience claim itself introduces specific challenges. The DT’s utility during an outage depends on the freshness of its state at the moment connectivity is lost: recently admitted patients may lack sufficient baseline history, and distribution shift between calibration and outage can degrade score accuracy precisely when it is most needed. The DT also introduces a new attack surface. Baseline poisoning during normal operation could produce a DT that appears healthy but systematically misprioritises patients during a subsequent outage.

Addressing these challenges will be essential for implementing DTs into HaH systems, ultimately leading to improved healthcare delivery and higher patient safety. Once those challenges are addressed, the implementation of DTs into existing and proposed HaH systems should be straightforward.

### Conclusions

The integration of DTs into HaH systems offers an approach to reduce cybersecurity-related risks for patients to a manageable level as required by current regulations, thus enhancing patient safety and system resilience. As sophisticated simulations of patients that are continually updated with real-time data, ward-level DTs can score the current risk, while patient-specific DTs will likely be able to predict the health trajectory of patients in the future, thus ensuring the integrity and availability of HaH services during cyberattacks or system failures. Our suggested approach of DT-enhanced HaH systems addresses critical concerns regarding patient safety related to service disruption, enabling healthcare providers to allocate resources efficiently and make informed decisions based on live risk scores just before the moment of system outage. This approach enhances the resilience and reliability of HaH systems, ensuring continuous and safe patient monitoring even in the face of cyber threats.

However, the implementation of DTs in HaH systems must overcome significant challenges, including ensuring data privacy, achieving interoperability across diverse data sources and types, and managing algorithmic biases. By addressing these challenges through robust security measures, transparent data management practices, and standardized assessment frameworks, this work provides a conceptual framework to inform future research and implementation of resilient HaH systems, offering high-quality and safe care in a digitally connected future healthcare system.

## Data Availability

No datasets were generated or analyzed during the current study.
